# Selections and Abstracts

**Published:** 1908-02

**Authors:** 


### SELECTIONS AND ABSTRACTS.

### RELATIONS BETWEEN CHRONIC GASTROINTESTINAL DISORDERS AND ANEMIC CONDITIONS.

Tixier concludes that insufficiency of the blood-forming function is not, primarily at least, at fault in the production of the secondary anemias following chronic gastrointestinal disease. He considers the anemia more probably due to the existence in the blood serum of hemolytic substances, perhaps produced by chemical changes connected with functional disturbances in the digestive organs. Besides the indirect pathologic evidence of the existence of such hemolytic action, he has been able to demonstrate it directly by the action of the serum of anemic rabbits on the blood of normal ones. With its globulicidal power, it has a certain stimulant action on the blood-forming apparatus in the bone marrow, which, however, seems to be more quickly exhausted than is its destructive action. His study, he considers, emphasizes the importance of functional over anatomic gastric lesions in the production of anemia. —*Semaine Medicale, June 19th, 1907.*

### RELATIVE VALUE OF OBSTETRIC FORCEPS AND VERSION AS A METHOD OF DELIVERY.

Wells summarizes his paper as follows:

"When rapid delivery is necessary, the pelvis being of normal size or flattened very slightly in the anteoposterior diameter, the transverse diameter being of normal size or increased, the cervix and vaginal canal dilated and well-softened version is preferable to forceps when mechanical interference is necessary. Among the special indications for version may be classed the following: Brow or face presentations with no rotation of the chin; certain cases of occipitoposterior position; parietal presentations; placenta praevia, if the presenting part is high up in the pelvis and the soft parts dilate slowly; certain cases of deficient uterine contractions; prolapse of the cord; transverse positions; excessively large caput succedaneum; some cases of eclampsia.

Forceps delivery gives best results in the largest number of labors delayed by weak uterine contractions in which interference is necessary, in the majority of cases of posterior rotation of the occiput. Forceps are also to be used in cases of rachitic, generally contracted, or in justominor pelves, providing the contraction is not great enough to prevent the head and shoulders of the child from passing through the inlet, and in those patients having certain constitutional diseases, when manual delivery is to be performed.

Neither forceps nor version should be attempted in any case of hydrocephalus or abnormally enlarged fetal body unless craniotomy or evisceration has first been performed."—*Therapeutic Gazette, Aug. '07.*

### LEGISLATION.

The following ridiculous laws have been introduced in the State Legislature of a Western State, according to the *Lancet-Clinic,* and shows the importance and necessity of physicians entering politics:

1. A law compelling all physicians to give duplicate prescriptions in English so that they can be read by anyone. Also giving in English, on prescription, name of trouble for which prescription was given.

2. A law compelling all physicians to give duplicate prescriptions, one for the druggist and one for the patient.

3. A law compelling the County Clerk to publish every three months a list of doctors who had deaths occur of patients under their charge, giving number of deaths after each name.

4. A law providing that where a physician prescribes morphine or opium for the cure or relief of a patient, and the patient acquires the habit therefrom, that charges may be brought against said physician, and upon conviction, he shall be sent to the penitentiary.

5. A law prohibiting a physician from charging more than the following scale of prices for calls: Between 7 a. m. and 6 p.m., $1; between 6 p. m. and 7 a. m., $1.50.

6. A law providing that should a patient not receive any benefit from a physician's treatment, that the physician can not collect his bill.

7. A law providing that in case of death of a patient while under the physician's care or treatment, bill for services must be canceled.

---

### *THE TREATMENT OF PUS TUBES.

BY JOHN EGERTON CANNADAY, M. D.,

Surgeon-In-Charge, Sheltering Arms Hospital, Hansford, W. Va.
Author's abstract from Lancet Clinic.

Treatment of pus tubes operative and non-operative. Again the cases to be treated are acute and chronic. The aim should be the restoration of function with the minimum disturbance, either anatomic or physiologic to the pelvic organs. Surgical sense and judgment are needed. The author advocates treating all acute cases conservatively and expectantly basing the treatment on the hope that every acute case may undergo resolution sufficient for the normal functionating of the pelvic organs. If restoration does not take place after several weeks of waiting, radical removal of the tubes is advocated. When this course is pursued, the results will be almost uniformly good. The author describes three clinical classes of pus tubes as follows; (1) Practically complete an early resolution a few fine adhesions and several tubal angulations remaining a few months after the attack. (2) The adhesions may be dense and powerful obliterating tubes and ovaries into a shapeless mass. (3) Large collections of pus are encapsulated and become sterile the tubes are virtually destroyed so far as any future function is concerned. The latter class gives the most positive results when surgically treated. During operation, the intestines should be well walled off with gauze and the pus aspirated; if there is danger of rupture and escape of pus, vaginal drainage is advocated. The author reports over 100 cases with a mortality of 1 per cent.

*Read before the Surgical Section of the Missipissippi Valley Medical Association Columbus Ohio, October 9 1807.

### THE IDEAL LIGATURE.

Whiting goes into the history of ligatures and sutures, before detailing his investigations in search of the ideal ligature, which he defines as one absolutely free from germ life, remaining sterile so long as it is retained in the tissues of the body, strong and flexible, and absorbed or replaced by living tissues after it has fulfilled its function. He considers that he has found such a ligature, and closes his article as follows: 1. The ideal ligature is obtainable by treating catgut before it has been twisted into a solid cord or string. 2. Catgut treated before it is twisted is much stronger than catgut sterilized after it has been made into a solid cord or string. 3. It is possible thoroughly to impregnate the untwisted gut with a chemical which will become a component part of the catgut when twisted. 4. Silver iodid gut will remain sterile as long as it is retained in the living tissues and will possess germicidal powers until it has been absorbed or replaced by living cells, the fluids of the body breaking up the silver iodid into silver, iodin, and various compounds of these chemicals. 5. It is possible and practicable to produce catgut that is absolutely free from germ life and that has antiseptic and germicidal powers, by treating the gut with various chemicals before it is twisted into a solid string or cord.—*New York Med. Journal, Sept. 14, 1907.*

### HEMOPTYSIS DUE TO TUBERCULOSIS.

Regarding this subject, Anders says: A retrospective view of the various methods of treatment employed during a comparatively brief period of time indicate clearly a notable diversity of opinion on the subject. It must be admitted also that certain well founded principles and facts are but dimly recognized by the present generation of physicians. For example, the bleeding from the hyperemic lung texture affords decided relief to the local pathologic condition, but more than this symptomatic improvement lasting weeks and months may be a secondary result. Again the profession has, it seems to me, an imperfect notion of the therapeutic value of absolute rest in this condition, as well as of approximately restricted feeding with light, unwarmed articles of diet.

Prophylactic measures of signal importance to the sufferer from hemoptysis are, I fear, systematically and inexcusably neglected by the mass of the profession. The avoidance of undue muscular exertion, of the free use of stimulants and strong mental excitement is to be strenuously enjoined. Certain climatic elements exercise a favoring influence and these are to be adversely considered in making a selection of a suitable habitat for the individual patient. Curtin long since pointed out that a residence far removed from the sea coast is best for patients suffering from hemoptysis, and further, "that rarefied, but also a cold, dry aseptic air would be most useful."

The remedial treatment is unsatisfactory and unpromising in view of the fact that, while numerous medicinal agents have been recommended, but few have given reliable evidence of their efficacy. It is in order that we put into force the few whose curative virtues have been proven. Unless there be obvious danger of inundation of the uninvolved lung tissue, as in cases of profuse hemorrhage, the cough should be arrested by the use of codein or, if troublesome, by morphin administered hypodermatically. It is necessary to stop disturbance of the bleeding point by coughing in order to give opportunity for the formation of a clot—Nature's own method of arresting hemorrhage. Hot drinks and alcoholic stimulants, if previously taken, must be intermitted.

My own best results during the early active stage of hemoptysis have been obtained from the strict enforcement of absolute recumbency and quiet, the patient not being allowed to speak aloud nor to put forth any muscular exertion, and an arrest of the cough by codein in the milder forms and morphin hypodermatically in the severer cases. The single contraindication to the use of opium has been pointed out, and if we accept the small class of cases in which it obtains, morphin or opium in some form, rightly administered, is the most servicable single remedy at our command; its signal virtue, I repeat, being ascribable to the remarkable enhancement of a coagulum at the seat of bleeding. I feel confident that the perils, near and remote, incident to the copious or otherwise protracted bleedings can be most successfully obviated by its judicious employment. My earnest plea is for a wider use and closer attention to the practical application of this sovereign agent. The importance of controlling the cough receives striking confirmation from the investigation of Dobell, previously mentioned. J. B. Walker also emphasized the value of opium in the treatment of hemoptysis in a paper read in 1889.

During the first twenty-four or forty-eight hours, according to the size of the hemorrhage, the patient should be kept well under its influence, the object being to stop the cough. Meanwhile the thorax must be carefully and frequently auscultated and should abundant, moist or bubbling rales be audible over the previously uninvolved portions of the lung—a rare event, except in case of rupture of a miliary aneurism—it is to be regarded as a signal for the withdrawal of the opiate.

While the arterial pressure in the lesser circulation is less than in the greater or the arterial system, it is subject to decided variations. The hypertrophy of the right ventricle which develops in the course of pulmonary tuberculosis it is fair to assume maintains an abnormally high degree of pressure in cardiopulmonary arc. This increased pressure may be also brought about by remedies that produce vaso-constriction in the peripheral vessels, such as ergot, and it is most probably accentuated by the forcible action of the heart in consequence of nervous excitation occasioned by hemorrhage. It is clear that a pure cardiac sedative, e.g., aconite, by lowering the tension in the lung vessels, must meet an important indication, and it may be prescribed with much confidence in its efficacy. I have found aconite to be highly serviceable in one-drop doses every third hour; it is especially useful in cases presenting fever. Lawrason Brown suggests "that the blood pressure be frequently observed, that morphin be used when necessary to quiet the patient and so equalize the blood pressure, that sodium nitrite be exhibited to reduce when necessary the blood pressure, and that in case of a sudden hemoptysis, amyl nitrite be administered at once when possible, to produce a marked fall in the blood pressure and so aid in a temporary cessation at least of the hemoptysis."

N. A. Johanson states that, in his experience, atropin in doses of gr. 1-50, repeated every twelve hours, has stopped hemorrhage when all other remedies have been of no avail. R. H. Babcock gives an immediate injection of atropin sulphate (gr. 1-50 to 1-25) when hemorrhage occurs from a cavity.

Successful treatment of the case in question must always be preceded by its reference to one or two categories previously mentioned, to-wit: (1) in which the hemoptysis is due to congestion or erosion of the smaller pulmonary vessels (most common); (2) in which there is profuse hemorrhage due to erosion of the larger pulmonary vessels or rupture of a small aneurism (comparatively rare).

The differentiation is greatly aided by remembering that the first class is composed principally of the early appearing cases, while the second class represents the cases which, in addition to showing profuse hemorrhage, occur at an advanced stage of this disease, as a rule. As stated in other connections in this article, the point of highest importance in the treatment of hemoptysis according to the nature of the lesion producing it, is that in profuse hemorrhage proceeding from one of the larger vessels or a ruptured aneurism, the cough is to be encouraged rather than stopped. Opiates are contraindicated.— *Jour. A. M. A., Sept. 28th, 1907.*

### PILOCARPIN AS AN ADJUVANT IN THE TREATMENT OF SYPHILIS.

William J. Robinson advocates the use of pilocarpin as an adjuvant to mercury in cases in which the system has become saturated with mercury and it has ceased to have the desired effect. The glands continue hard, swollen and tender, and stomatitis is marked. Gastrointestinal, hepatic, renal and skin activity have been neglected. The pilocarpin causes the elimination of mercury that was stored up in the salivary glands and acting as a toxic body. In the bowels and kidneys the elimination has been too great for the organs and pilocarpin causes the skin to act and carries the mercury to the seat of the skin lesions. The author recommends its use in doses of two to eight milligrams two or three times a day, without other drugs. It is of much value in secondary manifestations, and is a most remarkable glandular eliminant. In some cases supersaturation with mercury produces intolerance of the drug and the use of pilocarpin for a short time will enable us to resume mercury with excellent effect.—*Medical Record.*

### TREATMENT OF FRACTURES OF THE FEMUR.

E. M. Magruder, M. D., states that of all fractures, those involving the femur are the most difficult to manage. There are five evils liable to accompany these fractures, and five indications to be met in their treatment. There may be (1) shortening; (2) external rotation of the lower fragment due to loss of thigh rigidity; (3) backward angulation due to gravity induced by the dorsal position; (4) bed sores and hypostasis, and (5) general discomfort. To overcome these complications, the author employs a combination of Thomas' hip splint and Buck's extension apparatus. The essential points in the Thomas splint consist of an iron bar reaching from a point in the back two inches below the axilla to a point three inches above the malleoli, and three flexible iron bands riveted to the bar at right angles, a chest band, a thigh band and a leg band. The injured limb should be bathed with alcohol, and the trunk covered with a knit shirt worn between the skin and the splint. The Buck's extension is next applied, and the whole limb firmly and evenly padded with sheet wadding one-fourth inch thick and a bandage. The weights are applied and the foot of bed elevated. One anterior and two lateral coaptation splints are placed on the thigh and held on with adhesive strips. The Thomas splint is then applied and the iron bands for chest, thigh and leg adjusted. The whole limb is bandaged to the bar. The author claims this dressing meets all the indications of femoral fractures; shortening is prevented by the Buck's extension apparatus. External rotation is prevented by the firm application of the bandage the full length of the limb. Backward angulation is prevented by the broad longitudinal bar which runs down behind the seat of fracture. Bed sores and hypostasis are prevented by the ease with which a single nurse can, by turning the patient slightly to one side, effect a change of position and reach the back for alcohol massage. The comfort of the patient is surprising during the whole course of the treatment, and the bed pan can be used with the greatest facility. —*International Journal of Surgery.*

### A SIMPLE METHOD FOR THE PREVENTION OF POST OPERATIVE VOMITING.

One of the most annoying and often distressing complications of ether anesthesia is the persistent nausea and vomiting which often persists for some hours after an operation, and a method of treatment which will obviate post operative vomiting will surely be a great boon both to surgeons and their patients.

A recently advanced theory as to the cause of vomiting after ether, is that the stomach being kept empty for several hours before the anesthetic is given, during the operation the copious salivary secretion mixed with ether trickles down into the stomach and its irritating character sets up an inflammation of the gastric mucous membrane which produces vomiting. Acting upon this theory, MacArthur, of Winnipeg (Montreal Medical Journal, June 1907), conceived the idea that if the stomach was full of water at the time the anesthetic was given, the saliva surcharged with ether would be so largely diluted in the stomach that it could not irritate the mucous membrane. He reports 35 different operations with various periods of anesthesia, in nearly all of which there was a complete absence of vomiting.

In preparing a patient for a morning operation he advises a saline purge on the previous afternoon, light supper early in the evening; after midnight, 4 ounces of water every 4 hours, 2 hours before the operation 8 to 12 ounces of water and an additional 8 ounces half an hour before putting the patient on the table. He reports that anesthesia took place in all cases quickly and easily without any retching or vomiting while undergoing anesthesia or during the operation, and in nearly all the cases there was complete absence of post operative vomiting.

MacArthur found from his experience that the free administration of water previous to the anesthetic is of great service to the patient, that the intense thirst and retching usually present after ether anesthesia are absent, that the odor of the anesthetic is much more quickly eliminated from the system and that the patient is able to take food and enjoy it within six or eight hours of the operation.

The many advantages and the great comfort to the patient of preventing the post operative vomiting must comment this very simple method to all surgeons, and since no possible harm can follow it, we hope it will be thoroughly tested and if found successful that it will be universally employed.—Editorial, *St. Paul Med. Journal, Oct. 1907.*

---

### THE X RAYS IN GENERAL MEDICINE.

---

Pearson describes some of the many uses to which x rays may be put, and states that their value is greatly underestimated. Diagnosis: In cases of fracture and dislocation of bones, the x rays should be employed in all but the most simple cases. The taking of skiagrams in these cases is not a simple matter, and the correct interpretation of the shadows is still more difficult. The rays are of great value in the diagnosis of diseases of bones and joints—for example, in the distinctive diagnosis between tuberculosis, osteitis, periostitis, sarcoma and exostosis of bone. The distinctive diagnosis between gout and rheumatoid arthritis can usually be made with the help of the rays. In the chest, the employment of the rays for diagnostic purposes is most promising. In the abdomen the use of the rays is chiefly confined to the diagnosis of stone in the urinary tract. In the case of foreign bodies, the rays reveal not only the presence of the foreign body, but also its position. Therapeutics: The therapeutical effects of x rays may be classified as follows: X rays cause atrophy of the appendages of the skin. They stimulate the metabolism of tissues. They destroy certain pathological tissues. They possess marked anodyne effects. The possibility of their controlling or altering an opsonic index has yet to be settled. They possess no very definite action in destroying microorganisms. Tuberculous diseases respond most favorably to x rays, lupus vulgaris being the one lesion in which the most brilliant results are attained. Tuberculous glands and joints also respond favorably, but the results have not been satisfactory in the case of pulmonary tuberculosis.—*British Med. Journal, Sept. 14, 1907.*

### THE INDICATIONS FOR THERAPEUTIC UTERINE CURETTAGE.

Skeel condemns the indiscriminate use to which this time-honored and valuable operation is subjected. The slogan, "Here is a uterus, let's dilate and curette it," is altogether too prevalent. In incomplete abortion and in certain septic states of the endometrium, its use is, of course, entirely justified. Its employment in leucorrhea, however, is a very limited one indeed. In many instances the leucorrhea is merely a symptom of local congestion due to some general disturbance, such as anemia, neurasthenia, constipation, cardiac or pulmonary disturbance, which manifestly will not be improved by an operation. When the leucorrhea results from gonorrhea it is usually due to the involvement of the cervix. -Curettage of the cervix will only carry the infection to the body and will be of little avail anyhow. If the body is involved, curettage will only aggravate the condition and may bring about infection of the tubes. In dysmenorrhea, curettage avails little or nothing. In certain cases of stenosis of the internal os, dilation will relieve, but the relief only lasts from three to six months. In amenorrhea. curettage is absolutely useless. In sterility, it is only justifiable to curette when the husband is competent and there is no gross change in the tubes to account for the condition. Curettage has also been done too frequently for menorrhagia or metrorrhagia. If the bleeding is due to hemorrhagic endometritis and to polypi resulting from abortion, the curette is clearly indicated. There are many menorrhagias of the menopause which are not influenced by curettage. On the other hand, as an exploratory instrument its use may be eminently justified. As a palliative and preparatory measure in fibroids it is useful. Altogether the author believes that a large number of women who are now subjected to this so-called treatment are medical and not surgical patients.—*Cleveland Med. Jour.*, Sept.

### IS OPIUM USEFUL OR INJURIOUS IN CASES OF ACUTE PERITONITIS?

Professor Pel, of Amsterdam, recognizing the fact that formerly the opium treatment of acute peritonitis was the approved treatment, but that recently there had been a number of objections raised against it, proceeds to discuss the subject on its merits. He declares that it facilitates the rest in bed, which is so important, and also favors the localization of the morbid process by suppressing the peristaltic movements, which would break the adhesions. In order to succeed with the remedy, it should be given in one of the liquid preparations, such as laudanum, to which a few drops of brandy or of sherry may be added to avoid nausea. The medium dose would be five drops of laudanum every two hours, being guided by the intensity of the painful phenomena. It is proper to begin the treatment by an injection of morphine. Locally, he advises the application of an ice bag. It has been especially objected that the opium treatment favors intestinal paresis and the absorption of toxins; but the author considers this objection merely theoretical; he has never seen anything of this kind in his experience. Another objection stated that, as a result of the treatment, chronic constipation would result. This also is not valid, since opium really facilitates the evacuation of the contents when the crisis is over. The administration of laxatives, on the contrary, is likely to produce relapses. The opium treatment has one fault, and that is to mask the clinical picture. The appearance of favorable condition which it produces may deceive the physician and the patient; and the meteorism, which follows its use, makes it difficult to diagnosticate the symptoms relating to the peritonaeum. The author states that if small doses are used meteorism does not occur, since the peristaltic contractions are not paralyzed. Moreover, there are a number of clinical signs which are able to take the place of the impairment of the palpation by the meteorism, even should this be present. In about three-fourths of the cases, the immediate operation, during the acute stage, is not possible, and the best therapeutics consists then in absolute rest in bed, with a very low diet, the application of ice, and the administration of opium in suitable doses. It is absolutely necessary to refrain from administering laxatives.—*Berlin Klin Wock,* Aug. 12th, 1907, *N. Y. Med. Jour.*

---

### GENEROUS.

Mr. Meane—"I have nothing but praise for the new minister."

Mr. Goode—"So I noticed when the plate came around."

—*Philadelphia Inquirer.*

### SOME SILVER NITRATE POINTS VERY FREQUENTLY FORGOTTEN.

Nitrate of silver is too little appreciated by the general practician in throat troubles, especially in chronic laryngitis with probable involvement of the eustachian canal and consequent deafness. Liberal painting of the throat once a week, or at times more frequently, with a throat brush dipped in a watery solution of 10 grains of nitrate of silver to the ounce, will often induce a notable improvement. It does good, used in this manner, by contracting the overdistended blood vessels supplying the congested mucous membrane.

Nitrate of silver stains on the hands may be removed by applying a solution of potassium cyanide. The same agent will remove the discoloration on the skin of a patient, after the therapeutic effect is induced, thus permitting its use where it could not otherwise be employed.

Internally, in small doses, nitrate of silver stimulates the heart, tones the digestive functions when weakened by dyspepsia, promotes better nutrition, and acts as a nerve tonic. It may be given in distilled water, dispensed in dark bottle or with light excluding wrapper, and the caution to the patient to take it directly from the spoon without adding water before swallowing.

The local application of nitrate of silver solution to polypi of the ear will often induce their disappearance. It is used in strengths of 6 to 20 percent., regulating the frequency of application and the strength by the effect noted.

In chronic suppuration of the ear, nitrate of silver solution, in the strength of ¼ of 1 percent., may be dropped into the external meatus, after thorough cleansing. It frequently acts almost magically, though in some instances the strength may be increased even up to saturation.

A 10 percent. solution of silver nitrate, frequently applied, will often abort the distressing small boils seen in the external auditory canal of many patients of an exematous tendency.

Never use the solid stick or a large crystal of silver nitrate about the upper nares or throat. A small piece might become detached, and being swallowed, induce serious results.

A saturated solution of silver nitrate, painted over unbroken chilblains, will instantly relieve the itching and pain.

Bedsores may be aborted, even when the skin is very red and tender, by liberal painting with a 4 percent. solution of silver nitrate.—*Medical World,* Feb. '08.

---

### THERAPEUTIC VALUE OF APOMORPHIN HYDROCHLORID.

---

Fisk sums up his considerations of this subject practically as follows:

The effect of apomorphin hydrochlorid when administered by the mouth, is widely different from the hypodermic effect.

Hypodermically, it is a most valuable centric emetic in doses of from 1-20 to 1-6 grain, acting speedily, certainly and gently, even in cases of narcotic poisoning prior to the stage of coma. The average adult dose is 1-10 grain. He also recommended that it be tried in all cases in which hypnotics or antispasmodics are indicated, in doses preferably somewhat less than the emetic dose. depending on the tolerance of the individual, i. e., 1-40 grain or more.

When given hypodermically to children or debilitated subjects the possibility of its depressing effects should be borne in mind, and appropriate doses of strychnin simultaneously administered.

By the mouth, its centric effects are so uncertain as to render it useless as an emetic and of little value as a hypnotic. The effect is practically limited to expectorant action. The average adult dose is 1-8 grain every two or three hours, dissolved in syrup of wild cherry or syrup of lactucarium, with a few drops of dilute hydrochloric acid to insure solution.

It does not increase the effect of other narcotics, such as morphin, codein, or heroin, which may be simultaneously administered when it is desired to lower the excitability of the respiratory center without checking secretion. Strychnin may also be simultaneously administered in debilitated subjects for its stimulating effect on the respiratory center and to forestall possible depression; although even in the case of delicate children there is little fear of such depression from the administration of the pure crystaline preparation by the mouth.

Apomorphin, like other expectorants of its class, if used at an improper stage, when there is abundant secretion, or pushed to the extreme, may flood the bronchial tubes with mucus and drown the patient in his own secretion, especially if he lacks muscular power to expectorate. Such results, however, are not to be feared from an intelligent use of the remedy.

Crystalline apomorphin hydrochlorid should always be specified. There is a slight danger of adulteration with morphin if the drug is not thoroughly washed when manufactured. On general principles, the fresh preparation should be used if possible, but a greenish discoloration of tablets or solution does not necessarily contraindicate their use, especially if originally prepared from the pure crystalline salt by a reliable drug firm.—*Med. Record,* Sept 28, 1907.

### "KULIN POLYGAMY."

A practice that goes by this name—an absurd one, since there is no polygamy about it in the sense in which we understand it—has lately been the subject of several letters published in the London *Times,* as we learn from last Sunday's *Sun.* It seems to prevail among the Bengalis. Some of the *Times'* correspondents approve of it as a means of improving the physique of a race; others speak of it as "revolting" and "abhorrent." Among those who defend it is that interesting person Mr. George Bernard Shaw. It is well known of course that in most communities many women eligible to matrimony fail to form a marital alliance with a suitable man and consequently remain spinsters. Their reproductive capacity is wasted. The Bengal practice purports to spring from a patriotic desire to save this waste. Mr. Shaw says: "What does a Bengali father do under the same circumstances? He selects a picked man, a Brahman representing the highest degree of culture and character in his class, and pays him £700 to enable his daughter to become the mother of a well bred child."

There is no pretense of marriage, as we understand it; the Brahman is simply hired to beget a child. If more children are wanted, presumably other picked men may be provided for the same woman, and perhaps that accounts for the use of the word polygamy. The whole business seems to be on a par with the breeding of domestic animals. The farmer buys the service of a selected stallion for his mare; the rich Bengali hires a picked man to serve his daughter. We must agree with those who call the practice revolting and abhorrent. It is the effort of medical men to promote the physical welfare of all human beings, but never at the expense of morality and social order; consequently we expect that our British confreres will join us in protesting against approval of the strange Bengal custom.—*Ed. N. Y. Med. Jour.*, Oct. 12th, '07.

---

### URIC ACID SYMPTOMS AND THEIR RELIEF BY COLCHICUM AND THE SALICYLATES.

---

Leuf formulates our knowledge about uric acid symptoms and their relief by colchicum and the salicylates as follows: The aetiology of excessive uric acid formation is unknown, while the symptomatology of its excess is not fully known. The name rheumatism may generally be used synonymously with uric acid excess. The absence of uric acid evidence from the urine is no guarantee that it does not exist in abundance elsewhere in the body, and require elimination. It disturbs all the tissues of the body, though some much more than others, and is unusually selective of exceptional tissues in some cases, the reason for which is not understood. Only a single symptom may exist to call attention to even very large quantities signifying the eve of a severe affliction if not promptly eliminated. Many such conditions are not recognized, and are therefore unsuccessfully treated until the real condition is recognized. Symptoms that are benefited by colchicum or the salicylates are most probably due to uric acid, which causes irritative catarrh of the excretory mucosa, and probably most so as it nears the outer skin, thus affecting the bladder and urethra, as well as the enteric tract. Toothache of uric acid origin should not be overlooked, and is capable of prompt relief without the use of anodynes. There exists a visceral rheumatism, or pain and abnormal functioning of the viscera from uric acid disturbances, promptly relievable by colchicum or the salicylates and proper diet, and probably not by any other means. Uric acid symptomatology seems to be due to its accumulation in the tissues, because of its formation in excess of the ability of elimination; on this account structures poor in circulation are subject to its influence, as ligaments, bones, or serous membranes; or those in which, though their eliminative power is far greater than that of those just mentioned, the formation of waste products may be too rapid and bountiful to admit of prompt enough removal to prevent accumulation, as muscle. Wine of colchicum root is a remedy of remarkable efficiency for the relief of uric acid conditions, acting promptly in most instances, and the salicylates are valuable adjuncts to colchicum. The bowels should be kept freely open (not diarrhoeal) with magnesium sulphate, preferably small doses on arising, the amount depending upon the effect, one or two stools per day being desirable. Medicines should be pushed until effective, or until physiological evidences of their action ensue. All influences increasing circulation in or about the affected area are beneficial, such as heat, negative galvanism, the static breeze or spark, or warm bathing. As a rule all those medicinal agents are helpful which hasten elimination or increase oxidation, as the iodides, alkalies, iron and manganese.—*Medical Record,* Oct. 5th, 1907.

---

### THE UNBORN CHILD: ITS CARE AND ITS RIGHTS.

---

Helme says that we must recognize the rights of the unborn child to life; to protection from the hereditary taint of degeneracy; to health—that is, to conditions conducive to the safe-guarding of the health; to nature's food (when born)—that is, its mother's milk; to its natural protection (when born)—that is, its mother's care. The recognition of these rights demands the recognition of the following duties on the part of the parents, the profession and the State: 1. On the part of the parents: A clean and normal life before and after conception. 2. On the part of the mother: (a) The consistent regulation of her mode of life; (b) the abstention from alcohol; (c) the feeding of the child by the breast; (d) the care of the child after birth. 3. On the part of the medical profession: (a) The restriction of therapeutic feticide; (b) the education of a healthy public opinion. (c) On the part of the State: (a) Restriction of procreation to the fit, by the regulation and restriction of marriage to the fit, either by education or legislation and by the prevention of procreation by the unfit, whether by segregation of the unfit, or sterilization of degenerates, or the evolution of a healthy public opinion; (b) by regulation of the life of the pregnant woman by State provision of food for the necessitous, by provisions of hospitals for the reception of women during pregnancy, and by State prohibition of woman's work and employment during pregnancy; (c) immediate registration of stillbirths, premature labors, abortions, and, if practicable, pregnancies; (d) registration of birth within twenty-four hours; (e) regulation of the life of the woman who has given birth to a child by State provision of food for the necessitous, and by State prohibition of woman's work for at least six months after confinement.—*British Med. Jour.*

### PRECOCITY IN RELATION TO THE DUCTLESS AND ACCESSORY GENITAL GLANDS.

---

Guthrie reaches the following conclusions: Precocious physical development, sexual and somatic, may be due to tumors or to hypertrophy pituitary and pineal glands, of the adrenal cortex and accessory genital glands. The opposite condition—namely infantilism, or stunted growth, loss or nondevelopment of sexual attributes—may depend on atrophy or destruction of function of one or other of the organs named. Hypersecretion on the part of one or other of these glandular structures in the first case, and hypersecretion in the second, may account for the opposite conditions described. Precocious development (sexual and somatic) may in some cases be unassociated with any obvious lesion of glandular organs. There is reason to suppose that a correlation of function, or some mutual check action exists between the glands which have been mentioned. It is also probable that the thyreoid and thymus glands are intimately connected with the phenomena of physical and sexual development. Two distinct types of precocious development—namely, the obese and the muscular—are associated with tumors or hypertrophy of the adrenal cortex. The obese type may occur in both cases, but the muscular type seems to be confined to the male sex. Premature hirsuties occurs in practically all cases of premature physical development, but is not necessarily associated with other signs of sexual maturity. Tumors of the adrenal cortex which occasion precocious development are of mesothelial origin, and act by inducing multiplication and over growth, and therefore hypersecretion of the cerebral parenchyma cells.—*British Med. Jour.*, Sept. 21st, 1907.

---

A member of an eminent St. Louis law firm went to Chicago to consult a client. When he arrived he found that he had unaccountably forgotten the client's name He telegraphed his partner, "What is our client's name?"

The answer read, "Brown, Walter E. Yours is Allen, William B." —*Everybody's Magazine.*

### PRACTICAL DEDUCTIONS FROM AN EXPERIENCE OF 750 ABDOMINAL SECTIONS.

A. L. Smith sums up his paper as follows:

1. Operate on tubal pregnancy as soon as the condition is suspected. I lost one of my thirty-nine cases because I was not sure and waited until full time.

2. Operate on appendicitis as soon as the condition is suspected. Some of the cases which were just barely suspected proved on opening the abdomen to be gangrenous and even perforated.

3. Operate for cancer as soon as you suspect its presence; even then you will often be too late. If you wait until the diagnosis is absolutely certain, you will never be in time.

4. Operate for obstruction of the bowels as soon as vomiting becomes fecal, or sooner, if the condition is strongly suspected. Every case would be saved if the operation were done early enough.

5. Caesarian section would save all the mothers and nearly all the children who now die from eclampsia, placenta previa and contracted pelvis. If done early, it is a much safer operation than accouchement force, symphysiotomy or pubiotomy.

6. Every minute of anesthesia counts against the patient's life. Therefore operate as quickly as possible.

7. If the patient has been in hospital long enough to be well prepared, and by the aid of Trendelenburg posture, there will be no need to touch or even to see the intestines. Taking the bowels out of the abdominal cavity adds enormously to the danger of laparotomy.

8. We no longer lose patients from shock, which is really a mixture of prolonged anesthesia, exposure or handling of the intestines and profuse hemorrhage, for the simple reason that we avoid all these factors. Especially the last by tying all arteries before cutting them.

The lessons which my experience has taught me might also be summed up in the following don'ts:

Don't remove ovaries for dysmenorrhea until you have first tried dilation and curetting.

Don't operate on women who have just come in on the train. Every laparotomy case should be in bed in the hospital for three nights and two days before her operation.

Don't operate on enormously fat women. Spend a few months in reducing their weight first.

Don't remove pus tubes from very anemic women; their resisting powers are low. Spend a month in getting their blood improved.

Don't operate on patients with a high temperature, unless at the very beginning of the attack.

Don't do laparotomies on women whose lungs are tubercular.

Don't remove a cancerous uterus by the abdomen if you can do it by the vagina; if too far advanced to remove by the vagina, the liver and lymphatics are already saturated, and a curetting will do more good.

Don't be ashamed to back down when on opening the abdomen you find that a successful operation is impossible.

Don't keep a patient under an anesthetic much longer than an hour.

Don't lose an ounce of blood if you can save it by tying the arteries before you cut them.

Don't give more than three-quarters grain hypodermics; morphine causes pain from distension which is worse than the pain of the operation. —*Internat. Jour. of Surgery.*

---

Mark Twain knows that "it pays to advertise." At a recent dinner he said:

"When I was editing *The Virginia City Enterprise,* writing copy one day and mining the next, I tried in many ways to drive home the fact that advertising pays. One day I received a letter from a subscriber saying that he had found a spider pressed between the pages of his paper. He wanted to know whether this signified good or bad luck. I replied to him through our 'Answers to Correspondents' column as follows:

"Old Subscriber.—The finding of a spider in your copy of the *Enterprise* was neither good luck nor bad. The spider was merely looking over our pages to find out what merchant was not advertising in them, so that he could spin his web across his door and lead a free and undisturbed existence forever after."

*Everybody's Magazine.*

### INDIAN SUMMER.

BY DR. S. WEIR MITCHELL.

Slow falls the evening of the dying year,
Misty and dim the patient forests lie,
Chill ocean winds the wasted woodland grieve,
And earthward loitering the leaves go by.

The dead leaves rustle 'neath my lingering tread,
Low murmuring ever to the spirit ear;
We were, and yet again shall be once more,
In the sure circuit of the rolling year.

Trust thou the craft of nature. Lo! for thee
A comrade wise she moves, serenely sweet,
With wilful prescience mocking sense of loss
For us who mourn love's unreturning feet.

Trust thou her wisdom, she will reconcile
The faltering spirit to eternal change
When, in her fading woodways, thou shalt touch
Dear hands long dead and know them not as strange.

For thee a golden parable she breathes
Where in the mystery of this repose,
While death is dreaming life, the waning wood
With fair-caught light of heaven divinely glows.

Thou, when the final lonliness draws near,
And earth to earth recalls her tired child,
In the sweet constancy of nature strong
Shall dream again—how dying nature smiled.

*From The Century.*

### DANGER OF IT.

"Mother, mother, mother, turn the hose on me!" sang little Willie, as his mama was dressing him this morning.

"What do you mean?" she asked.

"You've put my stockin's on wrong side out," he said.

We fear Willie will grow up to be a newspaper humorist.

*—Cleveland Leader.*

### VERATRUM VIRIDE IN ECLAMPSIA.

Mirto gives the histories of sixty-one cases of eclampsia treated by the hypodermic use of extract of veratrum viride in the obstetrical clinics of Pavia and Milan. He recommends the use of this drug because the convulsions are invariably accompanied by a rise in arterial pressure, which may be regarded as an index of the approaching seizure, although it may not be the cause of the convulsions. He has observed that as soon as the arterial pressure is lessened the convulsions are moderated, the frequency of the attacks is lessened, and in some cases the convulsions are so controlled that the pregnancy can go on to full term and delivery be normal. He has also given it as a preventive measure in many cases in which there was albuminuria and casts accompanied by high arterial pressure, and believes that it prevented the convulsions which would otherwise have occurred. He recommends the use of the drug as a preventive and as a curative treatment for frequent and puerperal convulsions.—*Annali di Obstetrica E Ginecologia.*

---

A man entered a drug store in a hurry and asked for a dozen two-grain quinine pills.

"Shall I put 'em in a box, sir?" the clerk asked as he counted them out.

"Oh, no," replied the customer, "I want to roll them home."

*Everybody's*

---

As an illustration of woman's wit, Mr.Depew, who is still Senator from New York, cites the following:

A man once found that his wife had bought a few puffs of false hair. This displeased him. So one day he hid in the hall outside of her room, and, just as the lady was adjusting the false puffs, he darted in upon her.

"Mary," he said reproachfully, "why do you put the hair of another woman upon your head?"

"John," retorted Mary, with a glance at her husband's shoes, "why do you put the skin of another calf upon your feet?"

*Everybody's Magazine.*

